# Supplementary material for: Timing of Complementary Feeding, Growth, and Risk of Non-Communicable Diseases: Systematic Review and Meta-Analysis
Source: Nutrients. 2022 Feb 8;14(3):702. doi: 10.3390/nu14030702 (PMC8840757; doi:10.3390/nu14030702)
Supplement: Supplementary file 1 [file nutrients-14-00702-s001.zip › nutrients-1567691-Supplementary.pdf]

File S1. LITERATURE SEARCH AND SCREENING RESULTS

Key questions

- ***Does the onset of CF before 6 months of life lead to different nutritional and metabolic outcomes, in the short and long term, compared to exclusive breastfeeding for up to 6 months of age?***

- ***Does the onset of CF before 6 months of life lead to different nutritional and metabolic outcomes, in the short and long term, compared to feeding exclusively with infant formula or mixed (breastfeed + infant formula) for up to 6 months of age?***

PICOs

a.
P Healthy infant exclusively breastfed
I The onset of Complementary Feeding (CF) before 6 months completed (between 4 and 6 months of age)
C Compared to an onset of the Complementary Feeding (CF) at 6 months completed
O Different nutritional and metabolic outcomes in the short and long term

b.
P Healthy infant exclusively or predominantly fed with infant formula.
I The onset of Complementary Feeding (CF) before 6 months completed (between 4 and 6 months of age)
C Compared to an onset of the Complementary Feeding (CF) at 6 months completed
O Different nutritional and metabolic outcomes in the short and long term

KEY WORDS

Population

A. Infant
B. Child
C. [child]/lim
D. [infant]/lim

Exposure Factors / Comparison

<u>MeSH Terms/ Text word</u>: weaning; diet; food; infant; beverages; infant nutritional physiological phenomena; meals; food and beverages; infant food; eating; bottle feeding: bottle-fed; diet; diets; breast feeding

A. Feeding, Breast
B. Breastfeeding
C. Breast Feeding, Exclusive
D. Exclusive Breast Feeding
E. Breastfeeding, Exclusive
F. Exclusive Breastfeeding

G. Bottle feeding duration
H. Breast feeding duration
I. Solid food
J. Complementary feeding
K. Early weaning
L. Early complementary feeding
M. 'complementary feeding'/exp
N. 'weaning'/exp

Outcomes

A. Overnutrition
B. Obesity
C. Growth
D. Body Size
E. Body Height
F. Diabetes Mellitus
G. Noncommunicable Diseases
H. Nutritional and Metabolic Diseases
I. Pediatric Obesity
J. Overweight
K. Body Mass Index
L. Body Weight Changes
M. Body Weight
N. Body Composition
O. Nutritional Status
P. Growth and Development
Q. Fat body
R. Adipose tissue
S. Body fat
T. Adiposity rebound
U. 'obesity'/exp
V. 'body mass'/exp
W. 'overweight'
X. 'body weight'/exp
Y. growth
Z. 'growth'/exp
AA. 'adiposity rebound'/exp

Guidelines search

Temporal limitation: 2014-2021

PUBMED https://www.ncbi.nlm.nih.gov/pubmed/

#1

((((("Infant Nutritional Physiological Phenomena"[Mesh]) AND "Overnutrition"[Mesh]) OR "Growth"[Mesh]) OR "Diabetes Mellitus"[Mesh]) OR "Nutritional and Metabolic Diseases"[Mesh]) OR "Noncommunicable Diseases"[Mesh]

#2

(Complementary OR supplementary OR wean* OR transition* OR introduc* OR "Infant Nutritional Physiological Phenomena"[Mesh:noexp] OR weaning[mesh] OR ((Solid food*) OR solids)) OR “infant food”[mesh] OR infant feed*) AND (feeding* OR food* OR beverage*[tiab] OR beverages[mh] OR eating OR diet[tiab] OR diet[mh] OR meal*[tiab] OR meals[mh] OR "Food and Beverages"[Mesh] OR diets[tiab] OR “infant food”[mesh] OR infant feed* OR Bottle feeding[mh] OR bottle feeding*[tiab] OR bottle feeding OR bottle-feeding*[tiab] OR bottle-feedings OR bottle-fed[tiab] OR “bottle fed”[tiab] OR solid food

Filters activated: Guideline, Practice Guideline, published in the last 5 years.

#3

((((("Bottle Feeding"[Mesh]) OR "Breast Feeding"[Mesh]) OR "Infant Nutritional Physiological Phenomena"[Mesh]) OR "Weaning"[Mesh]) Or "Early weaning") OR "early introduction complementary feeding")

#4

((("Weaning"[All Fields]) OR "Infant Nutritional Physiological Phenomena"[MeSH]) OR "complementary feeding"[All Fields]) AND ((((((((((((("Obesity"[Mesh] OR "Pediatric Obesity"[Mesh]) OR "Overweight"[Mesh]) OR "Body Mass Index"[Mesh]) OR "Body Weight Changes"[Mesh]) OR "Body Weight"[Mesh]) OR "Body Composition"[Mesh]) OR "Nutritional Status"[Mesh]) OR "Growth and Development"[Mesh]) OR "Growth"[Mesh]) OR "fat body"[MeSH Terms]) OR "adipose tissue"[MeSH Terms]) OR body fat[Text Word]) OR “adiposity rebound” [Text Word]) AND "2014/05/08"[PDat]:"2021/08/14"[PDat] AND "infant"[MeSH Terms])

#5

(("weaning"[MeSH Terms] OR "weaning"[All Fields]) OR ("eating"[MeSH Terms] OR "eating"[All Fields]) OR "Feeding Behavior"[All Fields] OR "Complementary Feeding"[All Fields]) AND timing[All Fields] AND ("Growth and Development"[All Fields] OR ("growth and development"[Subheading] OR ("growth"[All Fields] AND "development"[All Fields]) OR "growth and development"[All Fields] OR "growth"[All Fields] OR "growth"[MeSH Terms]) OR "Nutritional Status"[All Fields] OR "Noncommunicable Diseases"[All Fields]) AND ((Practice Guideline[ptyp] OR Guideline[ptyp]) AND "2014/08/22"[PDat] : "2021/08/14"[PDat])

#6

("Nutritional Physiological Phenomena"[All Fields] OR "Infant Nutritional Physiological Phenomena"[All Fields] OR ("weaning"[MeSH Terms] OR "weaning"[All Fields])) AND ("Breast feeding"[All Fields] OR "Exclusive Breast Feeding"[All Fields] OR "bottle feeding"[All Fields] OR "formula feeding"[All Fields] OR "Exclusive bottle feeding"[All Fields] OR "Exclusive formula feeding"[All Fields]) AND ((timing[All Fields] AND ("food"[MeSH Terms] OR "food"[All Fields]) AND introduction[All Fields]) OR "Early infant feeding practice"[All Fields] OR "Early complementary feeding"[All Fields] OR (Timing[All Fields] AND ("food"[MeSH Terms] OR "food"[All Fields]) AND introduction[All Fields])) AND ("body composition"[All Fields] OR "fat mass"[All Fields] OR "Noncommunicable Disease"[All Fields] OR "Non Communicable Disease"[All Fields])

EMBASE https://www.embase.com

#1

(('bottle feeding'/exp OR 'bottle feeding' OR 'bottle feeding duration' OR 'breast feeding'/exp OR 'breast feeding' OR 'breast feeding duration'/exp OR 'breast feeding duration') AND ('weaning'/exp OR 'weaning' OR 'complementary feeding'/exp OR 'complementary feeding' OR 'early weaning' OR 'early complementary feeding')) AND ([2014-2021]/py AND ('practice guideline'/exp OR 'practice guideline' OR 'guideline'/exp OR guideline))

#2

l622344621/sim

#3

('complementary feeding'/exp OR 'complementary feeding' OR 'weaning'/exp OR weaning) AND ('obesity'/exp OR obesity OR 'body weight'/exp OR 'body weight' OR 'growth'/exp OR growth OR 'adiposity rebound'/exp OR 'adiposity rebound')

#3 AND (2014:py OR 2015:py OR 2016:py OR 2017:py OR 2018:py OR 2019:py OR 2020:py OR 2021:py) AND 'practice guideline'/de

UPTODATE https://www.uptodate.com/home

Society Guideline Links: *Breastfeeding and infant nutrition*

SOCIETY GUIDELINE LINKS: ***Complementary feeding, Weaning, Alimentary – nutrition, Breastfeeding, and Complementary Feeding***

National Guideline Clearinghouse (NGC) https://www.ahrq.gov/gam/index.html

Canadians Medical Association (CMA) https://www.cma.ca/clinicalresources/practiceguidelines

National Guideline Centre (NGC) - National Institute of Health and Care Excellence (NICE) https://www.rcplondon.ac.uk/about-us/what-we-do/national-guideline-centre-ngc

Scottish Intercollegiate Guidelines Network (SIGN) https://www.sign.ac.uk/our-guidelines.html

Australian Clinical Practice Guidelines (ACPG) https://www.clinicalguidelines.gov.au/

New Zealand Guidelines Group (NZGG) https://www.health.govt.nz/about-ministry/ministry-health-websites/new-zealand-guidelines-group

American Academy of Pediatrics (AAP) https://www.aap.org/en-us/Pages/Default.aspx
DateRange (01/01/2013-03/19/2021) AND ((complementary feeding) OR (weaning)) AND (Guideline)
North American Society for Pediatric Gastroenterology, Hepatology and Nutrition (NASPGHAN) https://www.naspghan.org/

European Society for Paediatric Gastroenterology Hepatology and Nutrition (ESPGHAN) http://www.espghan.org/

Geneva Foundation for Medical Education and Research (GFMER) https://www.gfmer.ch/Guidelines/Allattamento_it/Allattamento_alimentazione_complementare.htm

Società Italiana di Nutrizione Umana (SINU) http://www.sinu.it

Società Italiana di Pediatria (SIP) http://www-sip.it/

Società Italiana di Pediatria Preventiva e Sociale (SIPPS) https://www.sipps.it/

Società Italiana di Nutrizione Pediatrica (SINUPE) https://www.sip.it/2017/09/21/sinupe-societa-italiana-di-nutrizione-pediatrica/

Società Italiana di Endocrinologia e Diabetologia Pediatrica (SIEDP) http://www.siedp.it/pagina/84/linee+guida%2C+raccomandazioni+e+consensus

Sistematic Reviews (SRs) search

Temporal limitation: 2011-2021

PUBMED

#1

(Child Nutritional Physiological Phenomena OR Infant Nutritional Physiological Phenomena OR Weaning) AND (Breastfeeding OR Exclusive Breast Feeding OR bottle feeding OR formula feeding OR, Exclusive bottle-feeding OR Exclusive formula feeding) AND (timing food introduction OR Early infant feeding practice OR Early complementary feeding OR Timing of food introduction) AND (body composition OR fat mass OR Noncommunicable Disease OR non-communicable Disease)
Filters activated: Meta-Analysis, Systematic Reviews, Review, published in the last 10 years, Humans, Infant: birth-23 months

#2

systematic[sb] AND ((((("Infant Nutritional Physiological Phenomena"[Mesh]) AND "Overnutrition"[Mesh]) OR "Growth"[Mesh]) OR "Diabetes Mellitus"[Mesh]) OR "Nutritional and Metabolic Diseases"[Mesh]) OR "Noncommunicable Diseases"[Mesh])

Filters activated: Systematic Reviews, Meta-Analysis, published in the last 5 years, Child: birth-18 years.

#3

((((("Bottle Feeding"[Mesh]) OR "Breast Feeding"[Mesh]) OR "Infant Nutritional Physiological Phenomena"[Mesh]) OR "Weaning"[Mesh]) Or "Early weaning") OR "early introduction complementary feeding"

Filters activated: Systematic Reviews, Meta-Analysis, published in the last 10 years.

EMBASE

#1

('bottle feeding'/exp OR 'bottle feeding' OR 'exclusive breastfeeding'/exp OR 'exclusive breast feeding'/exp OR 'exclusive breastfeeding' OR 'exclusive breast feeding' OR 'exclusive bottle feeding' OR 'bottle feeding duration' OR 'breast feeding'/exp OR 'breast feeding' OR 'breast feeding duration'/exp OR 'breast feeding duration' OR 'weaning'/exp OR 'weaning' OR 'complementary feeding'/exp OR 'complementary feeding' OR 'early weaning' OR 'early complementary feeding') AND ([cochrane review]/lim OR [systematic review]/lim OR [meta analysis]/lim) AND [2011-2021]/py

#2

l622344621/sim

COCHRANE LIBRARY

#1

Child Nutritional Physiological Phenomena OR Infant Nutritional Physiological Phenomena OR Weaning) AND (Breastfeeding OR Exclusive Breast Feeding OR bottle feeding OR formula feeding OR, Exclusive bottle-feeding OR Exclusive formula feeding) AND (timing food introduction OR Early infant feeding practice OR Early complementary feeding OR Timing of food introduction) AND (body composition OR fat mass OR Noncommunicable Disease OR non-communicable Disease

#2

MeSH term – Weaning

#3

Phrase Matches - Infant Nutritional Physiological Phenomena

#4

MeSH descriptor – Obesity

publication date Between Jan 2011 and Aug 2021 (Word variations have been searched)

Studies search

PUBMED

#1

("Bottle Feeding"[Mesh] OR "Breast Feeding"[Mesh] OR "Infant Nutritional Physiological Phenomena"[Mesh] OR "Weaning"[Mesh] Or "Early weaning" OR "early introduction complementary feeding") AND ("Growth and Development"[All Fields] OR ("growth and development"[Subheading] OR ("growth"[All Fields] AND "development"[All Fields]) OR "growth and development"[All Fields] OR "growth"[All Fields] OR "growth"[MeSH Terms]) OR "Nutritional Status"[All Fields] OR "Noncommunicable Diseases"[All Fields]) AND "2015/08/22"[PDat] : "2021/08/14"[PDat])

#2

((("Weaning"[All Fields]) OR "Infant Nutritional Physiological Phenomena"[MeSH]) OR "complementary feeding"[All Fields]) AND ((((((((((((("Obesity"[Mesh] OR "Pediatric Obesity"[Mesh]) OR "Overweight"[Mesh]) OR "Body Mass Index"[Mesh]) OR "Body Weight Changes"[Mesh]) OR "Body Weight"[Mesh]) OR "Body Composition"[Mesh]) OR "Nutritional Status"[Mesh]) OR "Growth and Development"[Mesh]) OR "Growth"[Mesh]) OR "fat body"[MeSH Terms]) OR "adipose tissue"[MeSH Terms]) OR body fat[Text Word]) OR “adiposity rebound” [Text Word]) AND "2015/05/08"[PDat]:" 2021/08/14"[PDat] AND "infant"[MeSH Terms])

#3

(("weaning"[MeSH Terms] OR "weaning"[All Fields]) OR ("eating"[MeSH Terms] OR "eating"[All Fields]) OR "Feeding Behavior"[All Fields] OR "Complementary Feeding"[All Fields]) AND timing[All Fields] AND ("Growth and Development"[All Fields] OR ("growth and development"[Subheading] OR ("growth"[All Fields] AND "development"[All Fields]) OR "growth and development"[All Fields] OR "growth"[All Fields] OR "growth"[MeSH Terms]) OR "Nutritional Status"[All Fields] OR "Noncommunicable Diseases"[All Fields]) AND "2015/08/22"[PDat] : "2021/08/14"[PDat])

EMBASE

#1

('bottle feeding'/exp OR 'bottle feeding' OR 'bottle feeding duration' OR 'breast feeding'/exp OR 'breast feeding' OR 'breast feeding duration'/exp OR 'breast feeding duration') AND ('weaning'/exp OR 'weaning' OR 'complementary feeding'/exp OR 'complementary feeding' OR 'early weaning' OR 'early complementary feeding') AND ('clinical trial'/de OR 'cohort analysis'/de OR 'controlled clinical trial'/de OR 'cross-sectional study'/de OR 'longitudinal study'/de OR 'observational study'/de OR 'prospective study'/de OR 'randomized controlled trial'/de OR 'retrospective study'/de) AND (2019:py OR 2020:py OR 2021:py)

#2

('complementary feeding'/exp OR 'complementary feeding' OR 'weaning'/exp OR weaning) AND ('obesity'/exp OR obesity OR 'body weight'/exp OR 'body weight' OR 'growth'/exp OR growth OR 'adiposity rebound'/exp OR 'adiposity rebound') AND ('clinical trial'/de OR 'cohort analysis'/de OR 'controlled clinical trial'/de OR 'cross-sectional study'/de OR 'longitudinal study'/de OR 'observational study'/de OR 'prospective study'/de OR 'randomized controlled trial'/de OR 'retrospective study'/de) AND (2019:py OR 2020:py OR 2021:py)

COCHRANE LIBRARY

#1

(Infant Nutritional Physiological Phenomena) AND (growth OR obesity OR noncommunicable disease)

in Title Abstract Keyword - with Cochrane Library publication date Between Jan 2017 and Dec 2021 (Word variations have been searched)

#2

(Child Nutritional Physiological Phenomena OR Infant Nutritional Physiological Phenomena OR Weaning) AND (Breastfeeding OR Exclusive Breast Feeding OR bottle feeding OR formula feeding OR, Exclusive bottle-feeding OR Exclusive formula feeding) AND (timing food introduction OR Early infant feeding practice OR Early complementary feeding OR Timing of food introduction) AND (body composition OR fat mass OR Noncommunicable Disease OR non-communicable Disease)

In All Text

#3

Trials matching MeSH descriptor (explode all trees):
- Weaning
- Growth
- Body Size
- Pediatric Obesity
- Noncommunicable Diseases

range 2016-2021

#4

Phrase Matches - Any Word Match

- Infant Nutritional Physiological Phenomena

range 2016-2021

CLINICALTRIALS.GOV https://clinicaltrials.gov/

#1

Complementary feeding

Figure S1a: Guidelines search flow diagr

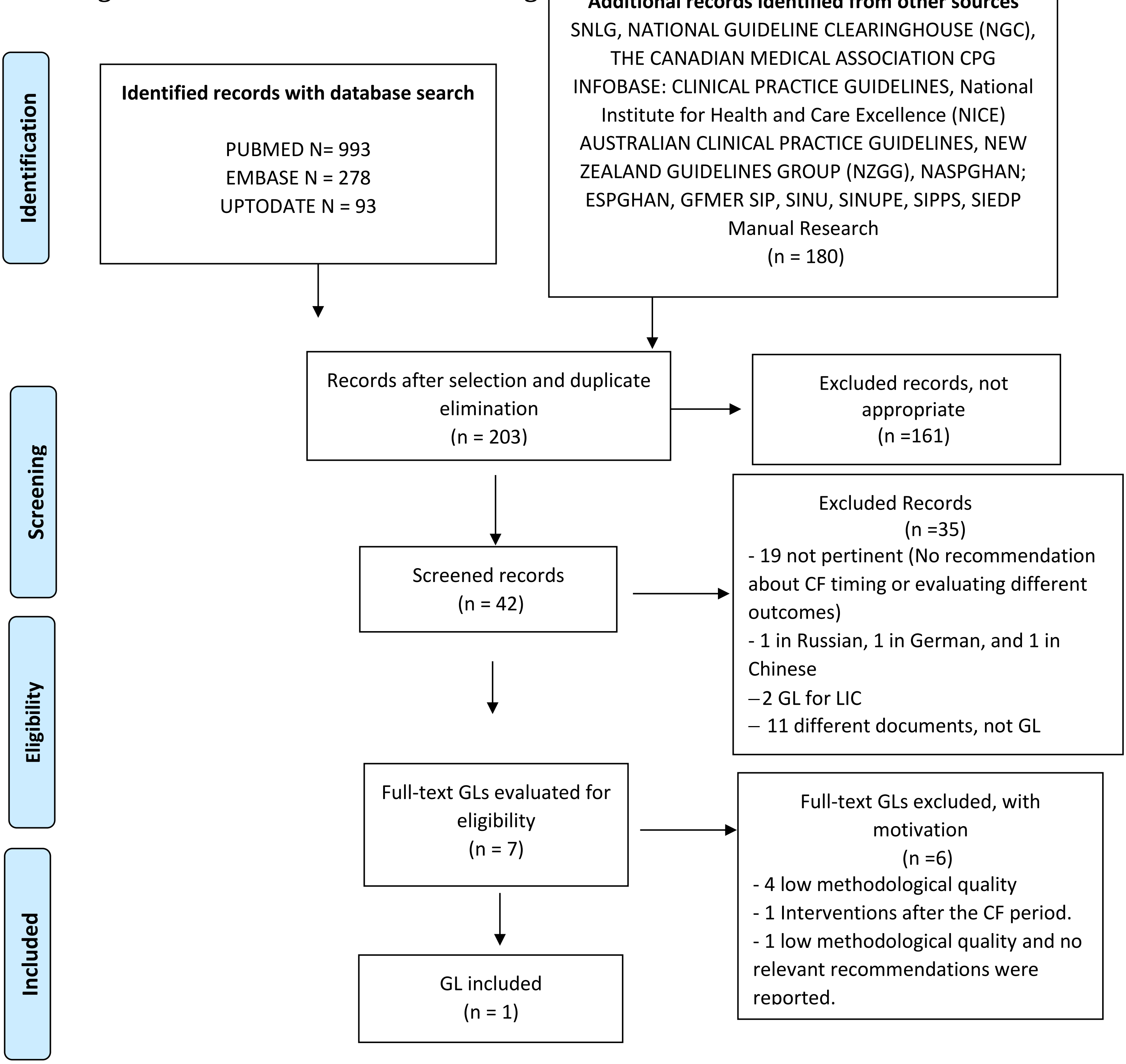

Figure S1b: SRs search flow diagram

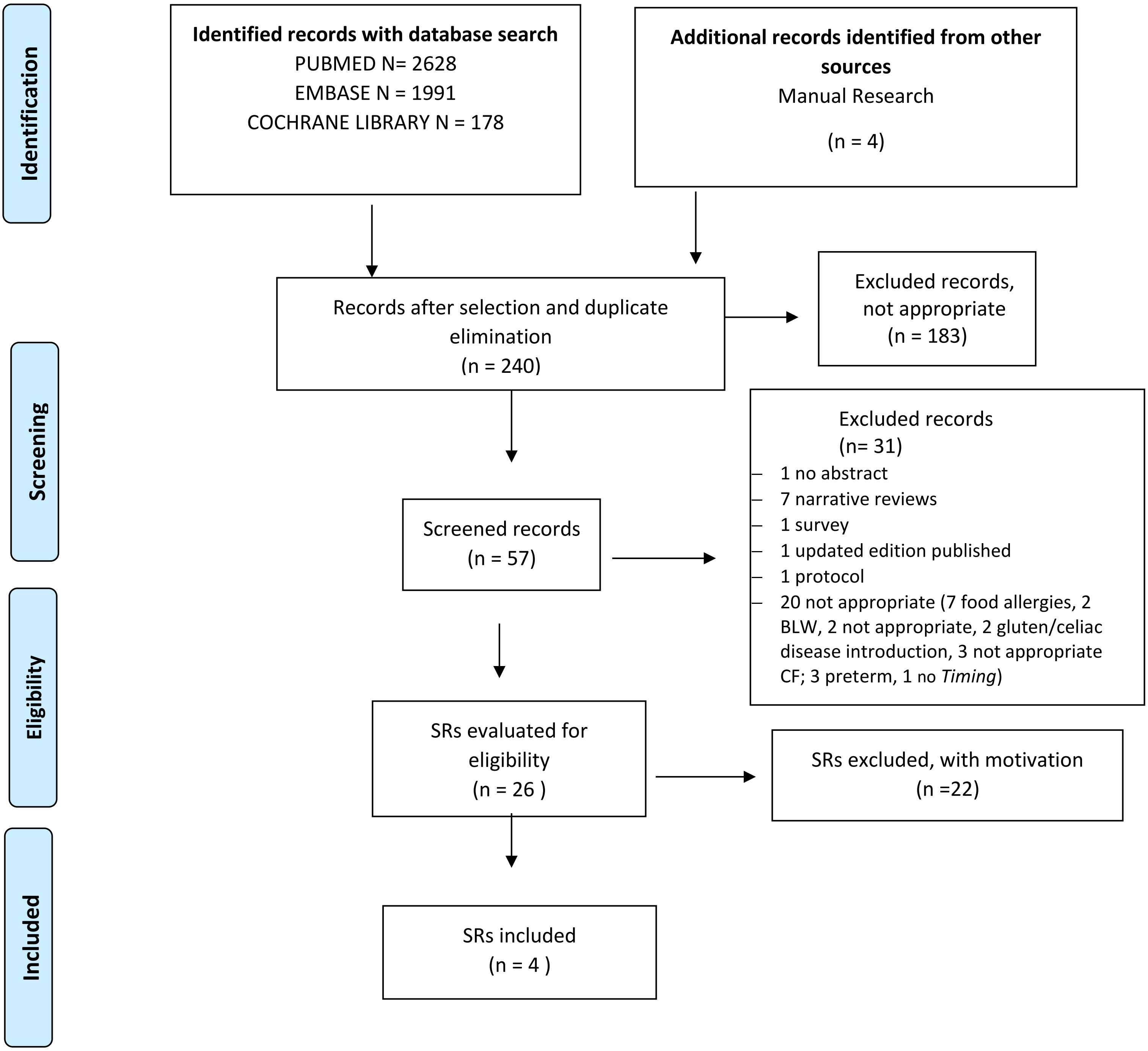

Figure S1c: Studies search flow diagram

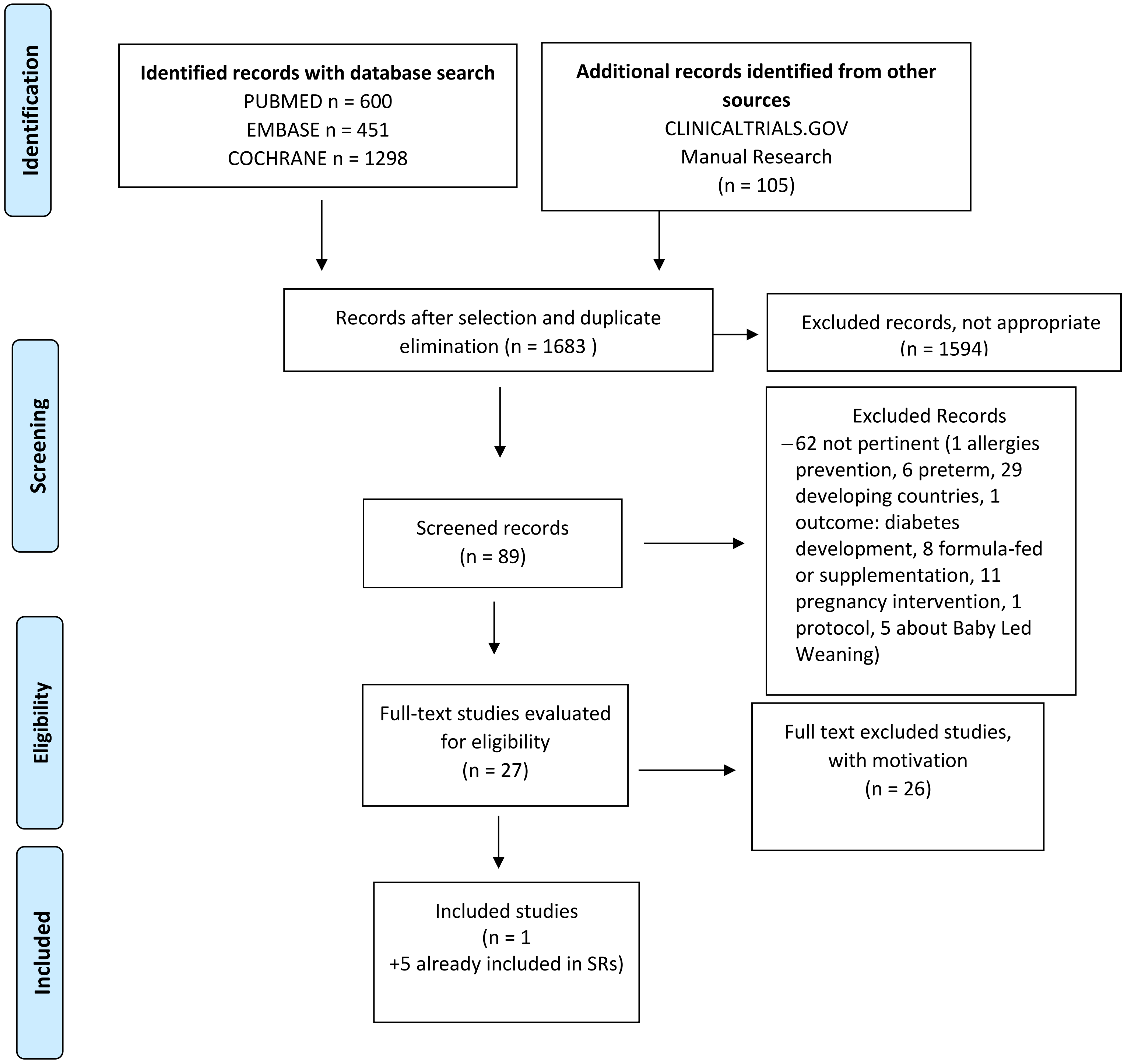

File S2. METHODOLOGICAL ASSESSMENT

Table S2a: Appraisal of the Clinical Guidelines and Documents

Guidelines and Clinical Guidance Documents	Methodological Evaluation			
	**Multidisciplinary panel**	**Systematic evidence research**	**Grading of recommendations**	**GL overall assessment**
SIEDP-SIP 2018 [1]	Yes	No, only MEDLINE	Yes	Moderate methodological quality.

Table S2b: Clinical Guidelines and Documents excluded.

GL Excluded	Multidisciplinary panel	Systematic evidence research	Grading of recommendations	Reason for exclusion
Alvisi et al. 2015 [2]	Limited to Pediatricians and Nutritionists.	No	No	Review document, with recommendations for clinical guidance.<br>Low methodological quality.
Fewtrell et al. 2017. ESPGHAN Complementary feeding [3]	No	Declared but not published.	No	Low methodological quality.
Davanzo et al. 2015. Breastfeeding [4]	No	No	No	Low methodological quality.
NICE 2015 Preventing excess weight gain [5]	===	====	===	Interventions after the CF period.
Romero-Velardea et al. 2016. Alimentation complementaria [6]	Limited To Pediatricians And Nutrition Experts.	No	No	Low methodological quality.
ACOG 2021 [7]	No	No	No	Low methodological quality. No relevant recommendations were reported.

Table S2c: Appraisal of the Systematic Reviews.

**AMSTAR 2**	Qasem et al. 2015 [8]	Smith et al. 2016 [9]	USDA et al. 2019 [10]	EFSA et al. 2019 [11]
**1. Did the research questions and inclusion criteria for the review include the components of PICO?**<br>**(Yes/No)**	No	Yes	Yes	Yes
**2. Did the report of the review contain an explicit statement that the review methods were established before the conduct of the review and did the report justify any significant deviations from the protocol?**<br>**(Yes/Partial Yes/No)**	Yes	Yes	Yes	Yes
**3. Did the review authors explain their selection of the study designs for inclusion in the review?**<br>**(Yes/No)**	Yes	Yes	Yes	Yes
**4. Did the review authors use a comprehensive literature search strategy?**<br>**(Yes/Partial Yes/No)**	Partial Yes	Partial Yes	Partial Yes	Partial Yes
**5. Did the review authors perform study selection in duplicate?**<br>**(Yes/No)**	Yes	Yes	Yes	Yes
**6. Did the review authors perform data extraction in duplicate?**<br>**(Yes/No)**	Yes	Yes	Yes	Yes
**7. Did the review authors provide a list of excluded studies and justify the exclusions?**<br>**(Yes/Partial Yes/No)**	Yes	Yes	Yes	Yes (Declared)
**8. Did the review authors describe the included studies in adequate detail?**	Yes	Yes	Yes	Yes

**(Yes/Partial Yes/No)**				
**9. Did the review authors use a satisfactory technique for assessing the risk of bias (RoB) in individual studies that were included in the review?**<br><br>**(Yes/Partial Yes/No/Includes only NRSI-RCT)**	Yes	Yes	Yes	Yes
**10. Did the review authors report on the sources of funding for the studies included in the review?**<br><br>**(Yes/No)**	No	Yes	No	No
**11. If meta-analysis was performed did the review authors use appropriate methods for statistical combination of results?**<br><br>**(Yes / No / No meta-analysis conducted)**	No Meta-Analysis Performed.	Yes	No Meta-Analysis Performed.	Yes
**12. If meta-analysis was performed, did the review authors assess the potential impact of RoB in individual studies on the results of the meta-analysis or other evidence synthesis?**<br><br>**(Yes / No / No meta-analysis conducted)**	No Meta-Analysis Performed.	Yes	No Meta-Analysis Performed.	Yes
**13. Did the review authors account for RoB in individual studies when interpreting/ discussing the results of the review?**<br><br>**(Yes/No)**	Yes	Yes	Yes	Yes
**14. Did the review authors provide a satisfactory explanation for, and discussion of, any heterogeneity observed in the results of the review?**<br><br>**(Yes/No)**	Yes	Yes	Yes	Yes
**15. If they performed quantitative synthesis did the review authors carry out an adequate investigation of publication bias (small study bias) and discuss its likely impact on the results of the review?**<br><br>**(Yes / No / No meta-analysis conducted)**	< 10 Studies In Meta-Analysis	< 10 Studies In Meta-Analysis	< 10 Studies In Meta-Analysis	< 10 Studies In Meta-Analysis

**16. Did the review authors report any potential sources of conflict of interest, including any funding they received for conducting the review?**<br><br>**(Yes/No)**	Yes	Yes	Yes	Yes
**OVERALL EVALUATION**	**MODERATE QUALITY**	**HIGH QUALITY**	**MODERATE QUALITY**	**MODERATE/HIGH QUALITY**
***** presence of 1 critical item and 2 failed non-critical items (n. 3, 15 e 16)				

Table S2d: SRs excluded with motivation.

SRs excluded	Reason for exclusion
Araújo et al. 2019 [12]	Timing of intervention and comparison not relevant (6-7 vs. 3-4 months)
Brown et al. 2019 [13]	No timing CF
Cordero et al. 2015 [14]	No timing CF
Daniels et al. 2015 [15]	Low methodological quality.
English et al. 2019 [16]	Same SR USDA 2019
Grabia et al. 2021 [17]	Low methodological quality. No timing CF.
Harrison et al. 2017 [18]	No timing CF
He et al. 2021 [19]	Not pertinent
Horta _WHO et al. 2013 [20]	No timing CF
Horta et al. 2015 [21]	Low methodological quality. No CF timing.
Horta et al. 2019 [22]	Low methodological quality.
Kramer et al. 2012 [23]	Timing of intervention and comparison not relevant (6-7 vs 3-4 months)
Martin et al. 2016 [24]	Intervention: 3 WHO recommendation.
Mathew et al. 2015 [25]	The section on anemia from RS Qasem 2015.
Mazarello Paes et al. 2015 [26]	No timing CF.
Moorcroft et al. 2011 [27]	Includes only one study already in most recent reviews (Kramer 1985).
Pearce et al. 2013 [28]	Timing of intervention and comparison not relevant
Qiao et al. 2020 [29]	methodological quality.
Spill et al. 2019 [30]	No timing CF
Vail et al. 2015 [31]	Low methodological quality.
Weng et al. 2012 [32]	Timing of intervention and comparison not relevant (<4 vs >4 mo)
Yan et al. 2014 [33]	The timing of intervention and comparison is not relevant.

Table S2e. Primary Studies Appraisal.

S2e. 1

	**Newcastle Quality Assessment Scale CASE-CONTROL STUDIES**								
	**Selection**				**Comparability**	**Exposure**			
**Study**	**Adequate case definition**	**Case Representativeness**	**Selection of Controls (community)**	**Definition of Controls (no outcome)**	**Comparability of cases and controls based on the design or analysis.**	**Ascertainment of exposure**	**The same method of ascertainment for cases and controls**	**Non-Response rate**	**Total**
Lopes et al. 2016 [34]	1a	1a	1a	1a	1+1 a,b	0c (Structured and validated questionnaires administered by experienced non-blinded personnel for cases and controls)	1a	0b	7

S2e.2

	**Newcastle Quality Assessment Scale COHORT STUDIES**								
	**Selection**				**Comparability**	**Outcome**			
**Study**	**Representativeness of the exposed cohort**	**Selection of the non exposed cohort**	**Ascertainment of exposure**	**Demonstration that outcome of interest was not present at the start of the study**	**Comparability of cohorts based on the design or analysis**	**Assessment of outcome**	**Was follow-up long enough for outcomes to occur**	**Adequacy of follow up of cohorts**	**Total**
Huh et al. 2011 [35]	1b	1a	1b	1a	1b	1b	1a	1a	8

S2e.3

	Newcastle Quality Assessment Scale STUDI CROSS-SECTIONAL STUDIES							
	**Selection**				**Comparability**	**Outcome**		
**Study**	**Representativeness of the sample**	**Sample size:**	**Non-respondents**	**Ascertainment of the exposure (max 2)**	**Comparability between groups, confounders are controlled (Maximum 2 stars)**	**Outcome evaluation (max 2)**	**Statistical test**	**Total**
Vail et al. 2015 [36]	1b	0	1	1	1	2	1	7

RCT

Figure S2a: Risk of bias summary: review authors' judgments about each risk of bias item for each included study.

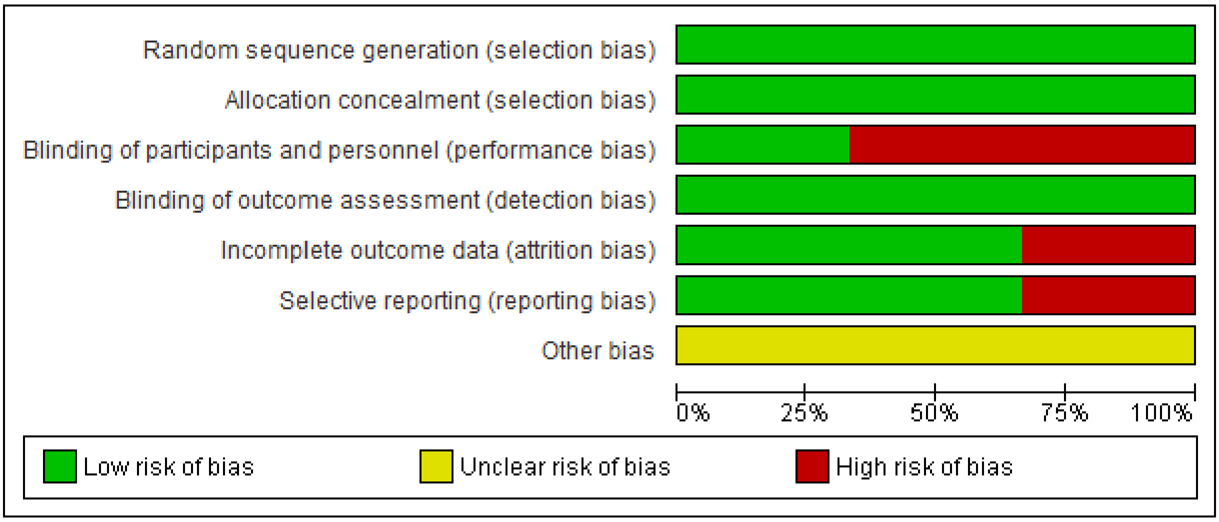

Figure S2b: Risk of bias graph: review authors' judgments about each risk of bias item presented as percentages across all included studies [37,38,39]

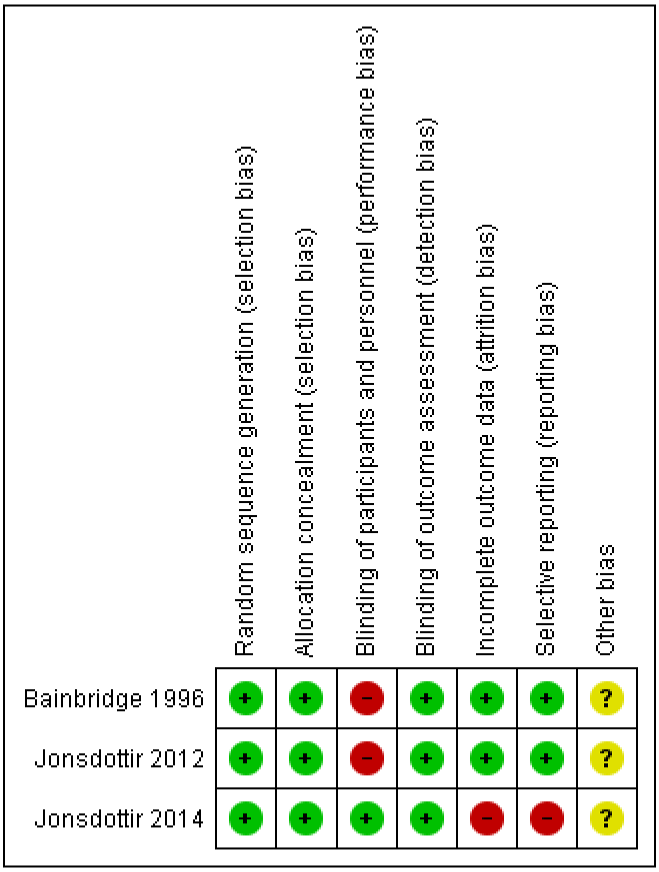

Table S2f: Excluded studies

Excluded studies	Reason for exclusion
Ardic et al. 2019 [40]	Timing CF intervention and/or comparison not pertinent.
Baldassarre et al. 2017 [41]	Timing CF intervention and/or comparison not pertinent.
Barrera et al. 2016 [42]	Low methodological quality: post hoc analysis, high dropout at follow-up.
Bell et al. 2018 [43]	Low methodological quality: post hoc analysis, 51% included in the final analysis, sample not representative of the original population.
Carruth et al. 2000 [44]	Low methodological quality, low sample size for multiple comparisons.
Carvalhaes et al. 2017 [45]	Not pertinent. Not assessing short and/or long-term outcomes.
Differing et al. 2020 [46]	Timing CF intervention and/or comparison not pertinent (< 4 vs > 4 mo).
Gingras et al. 2019 [47]	Low methodological quality (Loss to follow-up ~40%)
Grote et al. 2011 [48]	Low methodological quality (Loss to follow-up > 20%)
Horodynski et al. 2017 [49]	Timing CF intervention and/or comparison not pertinent.
Huus et al. 2008 [50]	Low methodological quality (Loss to follow-up > 50%. Self-reported data).
Mannan et al. 2018 [51]	Timing CF intervention and/or comparison not pertinent (< 4 vs > 4 mo).
Martin et al. 2017 [52]	Timing CF intervention and/or comparison not pertinent.
Morghen et al. 2018 [53]	Timing CF intervention and/or comparison not pertinent (< 4 mo).
Newby et al. 2015 [54]	Dietary Habits Survey. Not evaluating short and/or long-term outcomes.
Olaya et al. 2013 [55]	Not assessing the timing of the beginning of CF.
Olaya et al. 2017 [56]	Assesses surrogate outcomes (iron status).
Papotsou et al. 2018 [57]	Timing CF intervention and/or comparison not pertinent.
Pluymen et al. 2018 [58]	Timing CF intervention and/or comparison not pertinent.
Sandoval Jurado et al. 2016 [59]	Timing CF intervention and/or comparison not pertinent.
Schmidt Morgen et al. 2018 [60]	Timing CF intervention and/or comparison not pertinent.
Seach et al. 2010 [61]	Low methodological quality. Time intervals of CF initiation not specified, nor diet after 1 year of age. Loss to follow-up was not uniform between the 2 groups (those who completed the study had higher socioeconomic status and greater age).
Sirkka et al. 2018 [62]	Timing CF intervention and/or comparison not pertinent.
Trovão et al. 2020 [63]	Timing CF intervention and/or comparison not pertinent. (< 3 vs > 3 mo).
Usheva et al. 2021 [64]	Timing CF intervention and/or comparison not pertinent.
Wells et al. 2012 [65]	Same sample as Jonsdottir et al. 2012, different primary outcomes.

File S3. RECOMMENDATIONS OF THE GLs, RESULTS OF THE SRs AND STUDIES

a.<br>***Does the onset of CF before 6 months of life lead to different nutritional and metabolic outcomes, in the short and long term, compared to exclusive breastfeeding for up to 6 months of age?***	**P** Healthy infant exclusively breastfed<br>**I** The onset of Complementary Feeding (CF) before 6 months completed (between 4 and 6 months)<br>**C** Compared to an onset of the Complementary Feeding (CF) at 6 months completed<br>**O** Different nutritional and metabolic outcomes in the short and long term

Table S3a: Included Guidelines and other Documents: Recommendations and Grading

Guidelines – other Documents		Recommendations	Grading
SIEDP-SIP 2018 [1]	Diagnosis, Therapy, and Prevention of Obesity in Pediatric Age<br>(0-18 years)	**THERAPY**<br>- Care intervention of the first level is the responsibility of the family pediatrician.<br>The family pediatrician is in charge of individuating the children at risk of developing obesity<br>Condition.<br>Identification of effective early-life interventions targeting these modifiable factors is critical for obesity prevention.<br><br>Pediatrician task<br>To check beginning complementary feeding<br><br>**PREVENTION**<br>- Prevention of pediatric obesity is based, from the prenatal age, on the modification of dysfunctional behaviors (nutrition, physical activity, and sedentary lifestyle) which, by altering energy homeostasis, lead to excess weight.<br><br>- Exclusive breastfeeding until 6 mo is recommended.<br><br>- It is recommended that solids and liquids foods other than breast milk or infant formula are introduced no earlier than 4 mo and no later than 6 mo.	Level of evidence III, strength of recommendation A. Rif #310-311 (Sargent 2011, Daniels 2015)<br><br>Level of evidence I, strength of recommendation A.<br><br>Level of evidence III, strength of recommendation A Rif #344-347 Horta 2013, Yan 2014, Horta 2015)<br><br>Level of evidence III, strength of recommendation B Rif #348-352 (Pearce 2013, Weng 2012, Vail 2015, Seach 2010, Huh 2011)

Table S3b: Included SRs: Characteristics, Results, and Conclusions

Systematic Review	Population and purpose of the SR	Results	Conclusions

Qasem et al. 2015 [8]	Exclusively breastfed children between 4 and 6 mo.<br>Evaluate the scientific evidence and assess the relationship between the age of introduction of the CF with the iron asset and the growth in breastfed children.	Jonsdottir et al. 2012* – Growth 6 mo RCT _ n° 100 [Media (DS)]<br>Wt gain (z score): 6 m= -0.01(0.42); 4 m = -0.02(0.31); p= 0.90<br>Length gain (z score): 6m= 0.04 (0.51); 4m = 0.03 (0.50); p= 0.96<br>Wells et al. 2012* – growth 6 mo RCT– n° 100<br>Wt (z score): 6 mo= 0.36 (0.99); 4 mo= 0.28 (1.08); p= 0.7<br>Length (z score): 6 mo = 0.77 (0.84); 4 mo = 0.60 (0.92); p= 0.3<br>BMI (z score): 6 mo = -0.10 (1.04); 4 mo = -0.08 (1.14); p= 0.9<br>* *two articles from a single RCT*<br>Jonsdottir et al. 2012* – martial asset RCT _ n° 100<br>Hb: mean difference [MD]: 0.2 g/L; 95 % CI: -2.4, 2.8 g/L; p = 0.88<br>Ferritin: MD: 26.0 µg/L; 95 % CI: −0.1, 52.1 µg/L, p= 0.05	No significant difference has been found among the groups.<br>No significant difference has been found among the groups in terms of growth and body composition.<br>In infants of developed countries, the introduction of CF at 4 mo did not improve the martial asset compared to the introduction of CF at 6 mo.
Smith et al. 2016 [9]	Healthy children born to term and breastfed<br>Assess the benefits and risks of additional food or liquids; examine the introduction and type of additional food or liquids.	Jonsdottir et al. 2012* – Growth 6 mo RCT _ n° 100 (50+50)<br>Metanalysis Wt gain (z score): MD [95%IC]= -0.01[-0.15, 0.13]; p= 0.89	No significant difference has been found among the groups.
USDA et al. 2019 [10] (Research deadline on July-August 2016)	Infants and children generally healthy who have been fed with complementary food and drink from 0 to 24 mo<br>Evaluate growth, size, and/or body composition.<br>SR as part of the Pregnancy and Birth to 24 Mo Project (P/B-24 Project) by the Nutrition Evidence Systematic Review (NESR) team of the Center for Nutrition Policy and Promotion, Food and Nutrition Service, USDA.	Jonsdottir et al. 2012* – Growth 6 mo RCT _ n° 100<br>Wells et al. 2012* – Growth 6 mo RCT – n° 100<br>Jonsdottir, 2014 – RCT- overweight/obesity at 18, 28, and 38 mo<br>BMI-for-age at 18, o 29-38 mo,<br>WAZ at 18, or 29-38 mo<br>LAZ at 18, or 29-38 mo	No significant difference has been found among the groups.<br>Difference not significant
EFSA et al. 2019 [11] (Research deadline on May 2019)	Healthy infants or born preterm, or born small-for-gestational-age or with high growth velocity.<br>The appropriate age for introduction of complementary feeding of infants has been evaluated considering the effects on health outcomes, nutritional aspects, and infant development	Jonsdottir et al., 2014 - RCT- overweight/obesity at 29-36 mo<br>BMIZ CA 4 m vs. 6 mo: MD (z−score) [95%IC]= -0.15[-0.53, 0.23]<br>Huh et al., 2011 - Cohort- overweight/obesity at 3 years<br>BMIZ AC 4 m (n°= 427) vs. 6 mo (n°= 98): MD (z−score) [95%IC]= -0.06[-0.15, 0.27]<br>Odds obesity development at 3 years: OR [95%IC]= 0.28 [0.06; 1.25]	Difference not significant<br>Difference not significant<br>Difference not significant

Table S3c: Included studies: Characteristics and Results

Study (First Author, Year, Country/Setting)	Study design	Population (sample size, baseline characteristics)	Intervention/exposure and comparator	Primary Outcome	Effect measures	Secondary Outcomes	Follow-up	Results	Funding
Jonsdottir et al. 2012 [38]<br><br>7 Health Care Centers in Iceland	RCT	119 healthy term (≥37 weeks) singleton infants	complementary foods in addition to breast milk from 4 mo of age<br><br>compared with exclusively breastfed and complementary foods in addition to breast milk from 6 mo of age	Serum iron status at 6 months (Blood for hemoglobin (Hb), mean corpuscular volume (MCV), red blood cell distribution width (RDW), serum ferritin (SF), and total iron-binding capacity (TIBC))	Mean Difference (95% CI)	Gains (z-Scores) in infant weight, length, and head circumference during the study period	6 wks, and 3, 4, 5, and 6 mo	Data are mean (SD)<br><br>Serum iron status at 6 months in the 2 Intervention Groups, CF and EBF - N=94<br><br>Hb (g/L)<br>CF= 113.9 (6.1)<br>EBF= 113.7 (7.3): p=0,91<br><br>SF (mg/L)<br>CF = 70.0 (73.3)<br>EBF =44.0 (53.8); p= 0,02<br><br>Growth Rate in z Scores N=100<br><br>Wt gain from 0 to 6 mo<br>CF: - 0.55 (1,12);<br>EBF: - 0.46 (1,17); p=0,71<br><br>Length gain from 0 to 6 mo<br>CF: - 0.41 (0.95)<br>EBF: - 0.37 (1.18); p=0,85	Mead Johnson and the Eimskip Fund of the University of Iceland
Vail et al. 2015 CBGS Study [36]<br><br>Rosie Maternity Hospital in Cambridge, UK	Cross-sectional study	571 healthy infants, full-term birth (≥36 weeks); singleton birth	complementary foods from:<br>3.0-3.9 mo<br>4.0-4.9 mo<br>5.0-5.9 mo<br>6.0-6.9 mo	To test whether earlier age at weaning (age 3-6 mo) may promote faster growth during infancy<br>Anthropometric values were transformed into age- and sex-adjusted *z*-scores.<br><br>.	regression coefficient with zBMI		Birth, 3 mo and 12 mo	Age at weaning:<br>146 (25.6%) 4.0 - 4.9 mo,<br>226 (39.6%) 5.0 - 5.9 mo,<br>155 (27.1%) 6.0 - 6.9 mo<br><br>Exclusive BF N= 263<br><br>BMI z-score 12 mo – unstandardized regression coefficient;<br><br>Model 1: Adjusted for age, sex, maternal age, parity, and deprivation score 0.06 (-0.02 to 0.14) p=0.13<br><br>Model 2: Model 1 with additional adjustment for milk feeding at 3 mo. 0.02 (-0.06 to 0.11) p=0.56	Funded by European Union Framework V, World Cancer Research Foundation International (Ref 2004/03), Medical Research Council (Ref MC_UU_12015/2), Newlife Foundation (Ref 07/20), NIHR Cambridge Comprehensive Biomedical Research Center, and University of California San Francisco Pathway Explore Grant.
Jonsdottir et al. 2014 [39]<br><br>7 Health Care Centers in Iceland	RCT	119 healthy infant, Iceland, full-term birth (≥37 weeks); singleton birth	to receive complementary foods from the age of 4 mo in addition to breast milk (CF)	To test whether the duration of exclusive breastfeeding is protective of overweight and obesity later in life	BMI for age mean difference (95% CI)		Weight, length, and head circumference at 6 wks, 3, 4, 5, 6, 8, 10, 12,	No effects of exclusive breastfeeding for 4 or 6 mo on the growth pattern or the risk of being overweight or obese in early childhood<br><br>18 months of age<br>**CF EBF MD (95% CI) p-value**	Mead Johnson and the Eimskip Fund of the University of Iceland

			<u>compared</u> with to continue exclusive breastfeeding to the age of 6 mo (EBF)				and 18 mo and weight and height at 29–38 mo.<br><br>Data are presented as mean (SD<br><br>The risk of being overweight was defined as BMI-for-age >1 standard deviation (SD) above the WHO growth standard median. Overweight or obese was defined as a value >2 and >3 SDs, respectively, above the WHO growth standard median	BMI-for-age 0.60±0.92 0.59±0.95 0.009 (-0.38, 0.39) 0.96<br><br><u>29–38 months of age</u><br>BMI-for-age 0.64±0.86 0.79±0.83 -0.15 (-0.53, 0.24) 0.45	
Huh et al. 2011 [35]<br><br>Obstetrical offices of a multispecialty group practice in eastern Massachusetts	Cohort study	847 full-term infants,	Introduction of solid foods, categorized as:<br><br><4 mo,<br>4 to 5 mo,<br>and ≥6 mo.	obesity at 3 years of age (BMI for age and gender ≥95th percentile)	Odds of obesity (BMI ≥95th percentile), OR (95% CI)		3 y	Among formula-fed infants or infants weaned before the age of 4 mo, the introduction of solid foods before the age of 4 mo was associated with increased odds of obesity at age 3 years<br><br><u>Breastfed Age at Introduction of Solids</u>:<br><br><4 mo: N= 43<br>4-5 mo: N= 427<br>≥ 6 mo: N= 98<br><br><u>Odds of obesity (BMI ≥95th percentile), OR (95% CI)</u><br>Multivariable plus change in weight- for-age *z* score 0–4 mo<br><br><4 mo= 1 (0,3-4,4)<br>4-5 mo= 0,0 (Reference)<br>≥ 6 mo= 1 (0,4 to 2,5)	Funded by the National Institutes of Health (NIH).
Lopes et al. 2016 [34]<br><br>Public schools of Taubate (São Paulo, Brazil)	Case-control study	463 children	different ages of the introduction of CFs	To verify if in children in the early preschool age the prevalence of overweight and if introducing complementary	Correlation index with zBMI	To verify as the type of food introduced is associated with the prevalence of overweight	N.A.	The bivariate analysis and then linear regression analysis of multiple variables were conducted. The prevalence of overweight was elevated (27.5%).<br>Only birth weight showed a significant	not reported

				feeding is associated with this condition in this age group		in this age group		correlation to zBMI (r = 0.22, p < 0.0001)The early introduction of new foods is not a risk factor for the development of overweight at the beginning of preschool age.	

b.<br>***Does the onset of CF before 6 months of life lead to different nutritional and metabolic outcomes, in the short and long term, compared to feeding exclusively with infant formula or mixed (breastfeed + infant formula) for up to 6 months of age?***	**P** Healthy infant exclusively or predominantly fed with infant formula.<br>**I** The onset of Complementary Feeding (CF) before 6 months completed (between 4 and 6 months of age)<br>**C** Compared to an onset of the Complementary Feeding (CF) at 6 months completed<br>**O** Different nutritional and metabolic outcomes in the short and long term

Table S3d: Included Guidelines and other Documents: Recommendations and Grading

Guidelines – other Documents	Patients	Recommendations	Grading
SIEDP-SIP 2018 [1]	Diagnosis, Therapy, and Prevention of Obesity in Pediatric Age<br>(0-18 years)	**THERAPY**<br>- Care intervention of the first level is the responsibility of the family pediatrician.<br>The family pediatrician is in charge of individuating the children at risk of developing obesity<br>Conditions. Presence of risk factor: early complementary feeding…<br>Tasks. Control the age of onset of complementary feeding<br><br>**PREVENTION**<br>- Prevention of pediatric obesity is based, from the prenatal age, on the modification of dysfunctional behaviors (nutrition, physical activity, and sedentary lifestyle) which, by altering energy homeostasis, lead to excess weight.<br><br>- It is recommended that solids and liquids foods other than breast milk or infant formula are introduced no earlier than 4 mo and no later than 6 mo.	Level of evidence III, strength of recommendation A. Rif #310-311 (Sargent 2011, Daniels 2015)<br><br>Level of evidence I, strength of recommendation A.<br><br>Level of evidence III, strength of recommendation B Rif #348-352 (Pearce 2013, Weng 2012, Vail 2015, Seach 2010, Huh 2011)

Table S3e: Included SRs: Characteristics, Results, and Conclusions

Systematic Review	Population and purpose of the SR	Results	Conclusions

USDA et al. 2019 [10] (Research deadline on July-August 2016)	Infants and children generally healthy who have been fed with complementary food and drink from 0 to 24 mo<br><br>Evaluate growth, size, and/or body composition.<br><br>SR as part of the Pregnancy and Birth to 24 Mo Project (P/B-24 Project) by the Nutrition Evidence Systematic Review (NESR) team of the Center for Nutrition Policy and Promotion, Food and Nutrition Service, USDA.	Bainbridge, 1996 – RCT Growth at 26 weeks – n° 41<br><br>CFB at 16wk vs. EFF 16-26wk<br><br>Weight change NSGD (Difference not significant)<br><br>Length change at 26 weeks = NSGD; change 6-26 wk = 5.03, (SD 1.05) vs. 3.62 (SD:2.72), p=0.05.	Difference not significant
EFSA et al. 2019 [11] (Research deadline on May 2019)	Healthy infants or born preterm, or born small-for-gestational-age or with high growth velocity.<br><br>The appropriate age for introduction of complementary feeding of infants has been evaluated considering the effects on health outcomes, nutritional aspects, and infant development	Huh et al., 2011 - Cohort- overweight/obesity at 3 years<br><br>BMIZ CA 4 m (n°= 163) vs. 6 mo (n°= 25): MD (z−score) [95%IC]= -0.32[-0.74, 0.10<br>Odds obesity development at 3 years: OR [95%IC]= 1.00 [0.40; 2.50]	Difference not significant<br><br>Difference not significant

Table S3f: Included studies: Characteristics and Results

Study (First Author, Year, Country/Setting)	Study design	Population (sample size, baseline characteristics)	Intervention/exposure	Primary Outcome	Measures of treatment effect	Secondary Outcomes	Follow-up	Results	Funding
Bainbridge et al. 1996 [37]<br><br>University of Cincinnati Medical Center and 3 affiliated hospitals	RCT	41 healthy infant, full-term birth (37-41 wks), and appropriate for gestational age	The introduction of Rice cereal to the formula for 2 mo at 4 mo of age	To test whether intake of formula plus cereals between 16-26 wks (as compared to formula alone) would lead to lower bone mineral content, higher parathyroid hormone concentration, lower serum calcium, magnesium, and osteocalcin, and increased continuous night sleep	mean difference (p-value >/< 0,05)	differences of standard anthropometric measurements	2,4,5, and 6 mo	No significant differences between the two groups	Bristol-Myers Nutrition Center, University of Cincinnati, NIH HD 207489, NIH HD 11725, and NIH HD 07200
Huh et al. 2011 [35]<br><br>Obstetrical offices of a multispecialty group practice in eastern Massachusetts	Cohort study	847 full-term infants,	Introduction of solid foods, categorized as:<br><br><4 mo,<br>4 to 5 mo,<br>and ≥6 mo.	obesity at 3 years of age (BMI for age and gender ≥95th percentile)	Odds of obesity (BMI ≥95th percentile), OR (95% CI)		3 y	Formula-Fed Age at Introduction of Solids<br><br>Odds of obesity (BMI ≥95th percentile), OR (95% CI) Multivariable plus change in weight- for-age z score 0–4 mo<br><br><4 mo (n=91)= 6,3 (2,3 to 16,9)<br><br>4-5 mo (n=193)= 0,0 (Reference)<br><br>≥ 6 mo (n=25)= 3,6 (0,8 to 16,3)	NIH HD34568, HD64925, and HL68041.
Vail et al. 2015 CBGS Study [36]<br><br>Rosie Maternity Hospital in Cambridge, UK	Cross-sectional study	571 healthy infants, full-term birth (≥36 weeks); singleton birth	complementary foods from:<br>3.0-3.9 mo<br>4.0-4.9 mo<br>5.0-5.9 mo<br>6.0-6.9 mo	To test whether earlier age at weaning (age 3-6 mo) may promote faster growth during infancy Anthropometric values were transformed into age- and sex-adjusted *z*-scores.<br><br>.	regression coefficient with zBMI		Birth, 3 mo and 12 mo	Age at weaning:<br>146 (25.6%) 4.0-4.9 mo,<br>226 (39.6%) 5.0-5.9 mo,<br>155 (27.1%) 6.0-6.9 mo<br><br>Exclusive or partially FF No = 295<br><br>BMI z-score 12 mo – unstandardized regression coefficient<br><br>Model 1: Adjusted for age, sex, maternal age, parity, and	Funded by European Union Framework V, World Cancer Research Foundation International (Ref 2004/03), Medical Research Council (Ref MC_UU_12015/2), Newlife Foundation (Ref 07/20), NIHR Cambridge Comprehensive Biomedical Research

								deprivation score 0.06 (-0.02 to 0.14) p=0.13<br><br>Model 2: Model 1 with additional adjustment for milk feeding at 3 mo. -0.02 (-0.06 to 0.11) p= 0.56	Center, and University of California San Francisco Pathways Explore Grant.

[a] Multivariable model was adjusted for maternal education, household income, and prepregnancy BMI; paternal BMI; and child's age, gender, and race/ethnicity

File S4. META-ANALYSIS

Randomized Controlled Trials

Analysis 1.1 Weight z-score 6 months

	EBF 6 months			EBF 4-6 months				Mean Difference
**Study or Subgroup**	**Mean**	**SD**	**Total**	**Mean**	**SD**	**Total**	**Weight**	**IV, Fixed, 95% CI**
Jonsdottir 2012	-0.46	1.17	50	-0.55	1.12	50	100.0%	0.09 [-0.36, 0.54]
**Total (95% CI)**			**50**			**50**	**100.0%**	**0.09 [-0.36, 0.54]**
Heterogeneity: Not applicable								
Test for overall effect: Z = 0.39 (P = 0.69)								

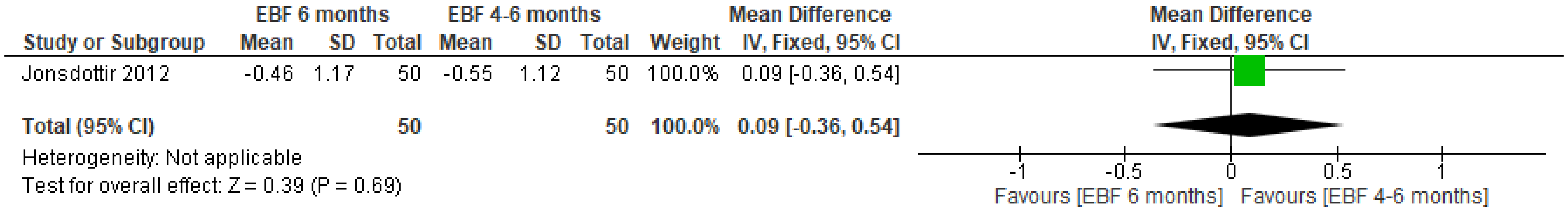

Analysis 1.2 Length z-score 6 months

	EBF 6 months			EBF 4-6 months				Mean Difference
**Study or Subgroup**	**Mean**	**SD**	**Total**	**Mean**	**SD**	**Total**	**Weight**	**IV, Fixed, 95% CI**
Jonsdottir 2012	-0.37	1.18	50	-0.41	0.95	50	100.0%	0.04 [-0.38, 0.46]
**Total (95% CI)**			**50**			**50**	**100.0%**	**0.04 [-0.38, 0.46]**
Heterogeneity: Not applicable								
Test for overall effect: Z = 0.19 (P = 0.85)								

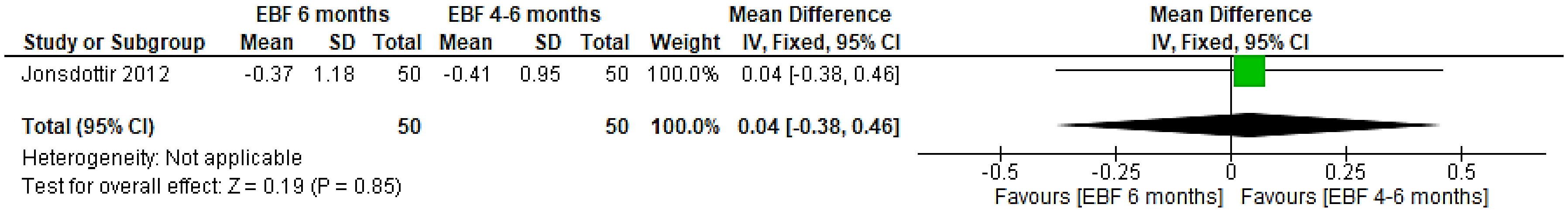

Analysis 2.1 % Overweight 18 months of age

Study or Subgroup	EBF 6 months Events	EBF 6 months Total	EBF 4-6 months Events	EBF 4-6 months Total	Weight	Risk Ratio M-H, Fixed, 95% CI
Jonsdottir 2014	5	46	4	48	100.0%	1.30 [0.37, 4.56]
**Total (95% CI)**		**46**		**48**	**100.0%**	**1.30 [0.37, 4.56]**
Total events	5		4			

Heterogeneity: Not applicable
Test for overall effect: Z = 0.42 (P = 0.68)

Risk Ratio
M-H, Fixed, 95% CI

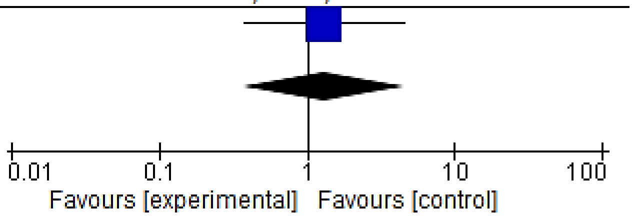

Analysis 2.2 BMI for age 18 months

Study or Subgroup	EBF 6 months Mean	EBF 6 months SD	EBF 6 months Total	EBF 4-6 months Mean	EBF 4-6 months SD	EBF 4-6 months Total	Weight	Mean Difference IV, Fixed, 95% CI
Jonsdottir 2014	0.59	0.95	46	0.6	0.92	48	100.0%	-0.01 [-0.39, 0.37]
**Total (95% CI)**			**46**			**48**	**100.0%**	**-0.01 [-0.39, 0.37]**

Heterogeneity: Not applicable
Test for overall effect: Z = 0.05 (P = 0.96)

Mean Difference
IV, Fixed, 95% CI

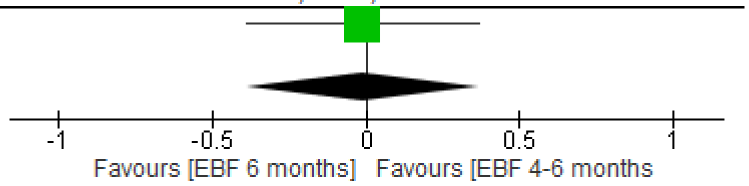

Observational studies

Analysis 3.1 Weight z-score 12 months

Age at weaning: mo 4.0-4.9 vs 6.0-6.9

	CF 6 months			CF 4-6 months				Mean Difference	Mean Difference
**Study or Subgroup**	**Mean**	**SD**	**Total**	**Mean**	**SD**	**Total**	**Weight**	**IV, Fixed, 95% CI**	**IV, Fixed, 95% CI**
Vail 2015	0.25	0.92	155	0.58	0.99	146	100.0%	-0.33 [-0.55, -0.11]	
Vail 2015	0.25	0.92	155	0.39	0.95	226	0.0%	-0.14 [-0.33, 0.05]	
**Total (95% CI)**			**155**			**146**	**100.0%**	**-0.33 [-0.55, -0.11]**	
Heterogeneity: Not applicable									
Test for overall effect: Z = 2.99 (P = 0.003)									

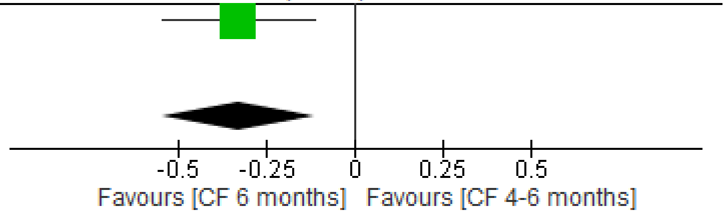

Age at weaning: mo 5.0-5.9 vs 6.0-6.9

	CF 6 months			CF 4-6 months				Mean Difference	Mean Difference
**Study or Subgroup**	**Mean**	**SD**	**Total**	**Mean**	**SD**	**Total**	**Weight**	**IV, Fixed, 95% CI**	**IV, Fixed, 95% CI**
Vail 2015	0.25	0.92	155	0.58	0.99	146	0.0%	-0.33 [-0.55, -0.11]	
Vail 2015	0.25	0.92	155	0.39	0.95	226	100.0%	-0.14 [-0.33, 0.05]	
**Total (95% CI)**			**155**			**226**	**100.0%**	**-0.14 [-0.33, 0.05]**	
Heterogeneity: Not applicable									
Test for overall effect: Z = 1.44 (P = 0.15)									

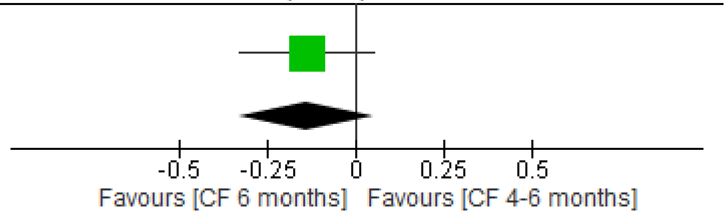

Analysis 3.2 Length z-score 12 mesi

Age at weaning: mo 4.0-4.9 vs 6.0-6.9

	CF 6 months			CF 4-6 months				Mean Difference	Mean Difference
Study or Subgroup	Mean	SD	Total	Mean	SD	Total	Weight	IV, Fixed, 95% CI	IV, Fixed, 95% CI
Vail 2015	0	1.04	155	0.48	1.05	146	100.0%	-0.48 [-0.72, -0.24]	
Vail 2015	0	0	155	0.23	1.04	226		Not estimable	
**Total (95% CI)**			**155**			**146**	**100.0%**	**-0.48 [-0.72, -0.24]**	
Heterogeneity: Not applicable									
Test for overall effect: Z = 3.98 (P < 0.0001)									

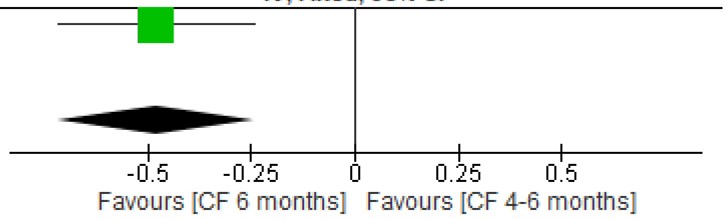

Age at weaning: mo 5.0-5.9 vs 6.0-6.9

	CF 6 months			CF 4-6 months				Mean Difference	Mean Difference
Study or Subgroup	Mean	SD	Total	Mean	SD	Total	Weight	IV, Fixed, 95% CI	IV, Fixed, 95% CI
Vail 2015	0	1.04	155	0.48	1.05	146	0.0%	-0.48 [-0.72, -0.24]	
Vail 2015	0	1.04	155	0.23	1.04	226	100.0%	-0.23 [-0.44, -0.02]	
**Total (95% CI)**			**155**			**226**	**100.0%**	**-0.23 [-0.44, -0.02]**	
Heterogeneity: Not applicable									
Test for overall effect: Z = 2.12 (P = 0.03)									

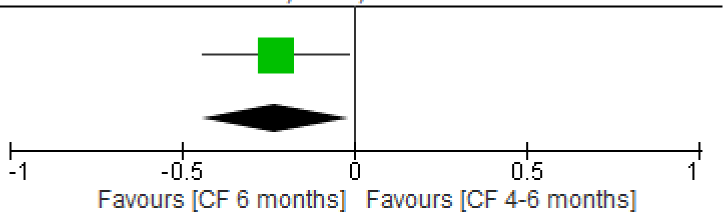

Analysis 4.1 Overweight/Obesity 3-6 years (Breast-Fed)

Study or Subgroup	EBF 6 months Events	EBF 6 months Total	EBF 4-6 months Events	EBF 4-6 months Total	Weight	Risk Ratio M-H, Random, 95% CI
Huh 2011	19	98	104	427	100.0%	0.80 [0.51, 1.23]
**Total (95% CI)**		**98**		**427**	**100.0%**	**0.80 [0.51, 1.23]**
Total events	19		104			
Heterogeneity: Not applicable						
Test for overall effect: Z = 1.02 (P = 0.31)						

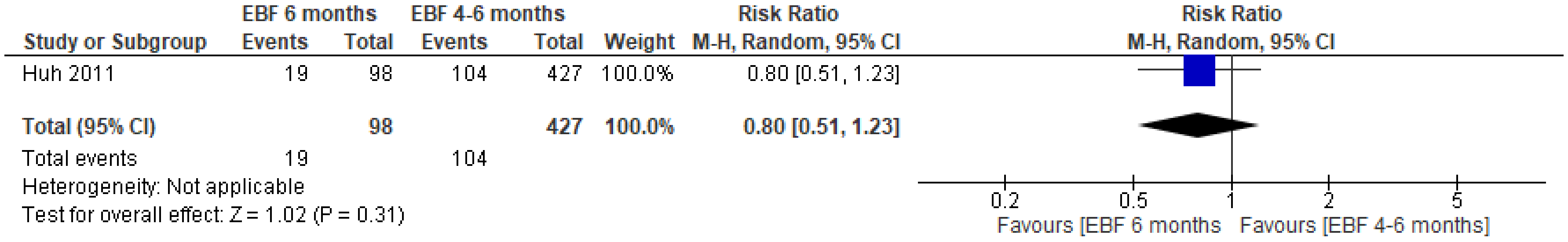

Analysis 4.2 Overweight/Obesity 3-6 years (Formula Fed)

Study or Subgroup	EFF 6 months Events	EFF 6 months Total	EFF 4-6 months Events	EFF 4-6 months Total	Weight	Risk Ratio M-H, Fixed, 95% CI
Huh 2011	8	25	42	163	100.0%	1.24 [0.66, 2.33]
**Total (95% CI)**		**25**		**163**	**100.0%**	**1.24 [0.66, 2.33]**
Total events	8		42			
Heterogeneity: Not applicable						
Test for overall effect: Z = 0.68 (P = 0.50)						

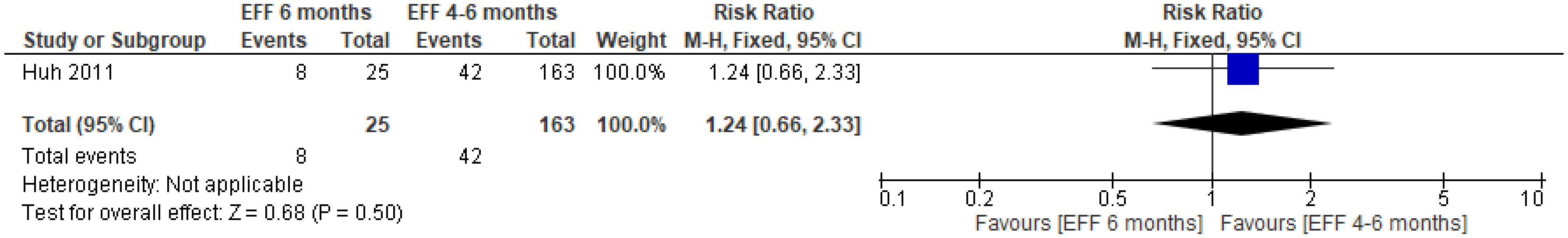

Iron Status Indices

Analysis 5.1 Hb (g/dl) 6 months

Study or Subgroup	EBF 6 months Mean	SD	Total	EBF 4-6 months Mean	SD	Total	Weight	Mean Difference IV, Fixed, 95% CI
Jonsdottir 2012	11.4	7.3	49	11.4	6.1	49	100.0%	0.00 [-2.66, 2.66]
**Total (95% CI)**			**49**			**49**	**100.0%**	**0.00 [-2.66, 2.66]**

Heterogeneity: Not applicable
Test for overall effect: Z = 0.00 (P = 1.00)

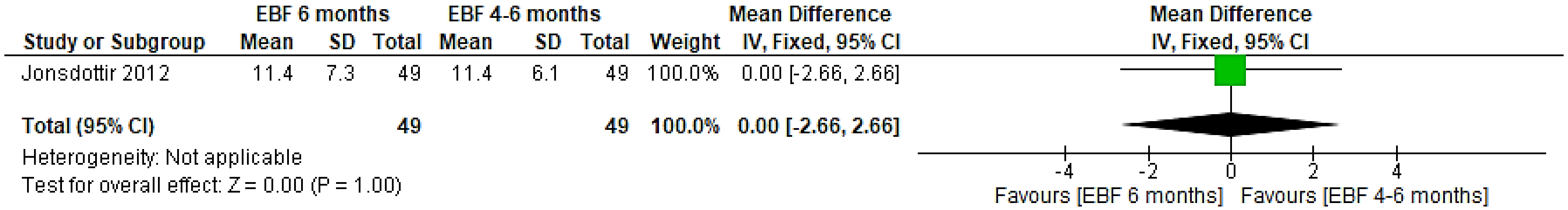

Analysis 5.2 Serum Ferritin (μg/L) 6 months

Study or Subgroup	EBF 6 months Mean	SD	Total	EBF 4-6 months Mean	SD	Total	Weight	Mean Difference IV, Fixed, 95% CI
Jonsdottir 2012	44	53.8	47	70	73.3	47	100.0%	-26.00 [-51.99, -0.01]
**Total (95% CI)**			**47**			**47**	**100.0%**	**-26.00 [-51.99, -0.01]**

Heterogeneity: Not applicable
Test for overall effect: Z = 1.96 (P = 0.05)

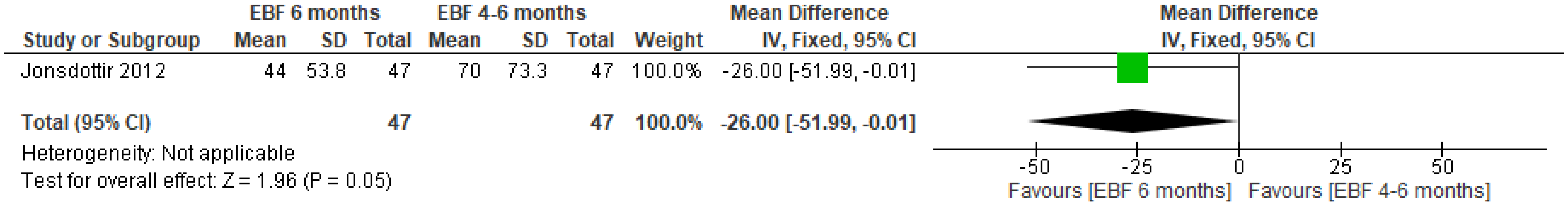

File S5. SUMMARY OF FINDINGS FOR THE MAIN COMPARISONS

Table S5a: Growth. Blood iron status

[Introduction CF at 4-6 month] than [Introduction CF at 6 months] in order to [ensure adequate growth at 6-12-18-24 months]

Patient or population: [ensure adequate growth at 6-12-18-24 months]

Setting: Outpatient

Intervention: [Introduction CF at 4-6 month]

Comparator: [Introduction CF at 6 month]

outcomes	**Anticipated absolute outcome**[*] (95% CI)		Relative outcome (95% CI)	№ of participants (studi)	Certainty of evidences (GRADE)	Comments
	**Risk with [Introduction CF at 4-6 month]**	**Risk with [Introduction CF at 6 month]**				
Weight gain Z-score (WGZ)<br>follow up: average 6 months	The average weight gain Z-score was = -0.01	MD = **-0.01**<br>(0.15 inferior to 0.13 major)	-	141<br>(2 RCT) [1,2,,a]	⊕⊕⊕◯<br>MODERATE [b]	
Length gain Z-	The average length gain Z-	MD = **-0.01**<br>(0.21 inferior to 0.19 major)	-	141<br>(2 RCT) [1,2,a]	⊕⊕⊕◯<br>MODERATE [b]	

score (LGZ)<br>follow up: 6 months	score was **= -** 0.01				
Serum Hb (Hb)<br>evaluated with: gr/L<br>follow up: 6 months	The average serum hb was **=** 0.2	MD **= 0.2**<br>(2.44 inferior to 2.48 major)	-	100<br>(1 RCT) [1]	⊕⊕⊕◯<br>MODERATE [c]
Serum Ferritin (SF)<br>evaluated with: ug/L<br>follow up: 6 months	The average serum ferritin was **=** 26	MD = **26**<br>(0.1 inferior a 52.1 major)	-	100<br>(1 RCT) [1]	⊕⊕⊕◯<br>MODERATE [c]
Weight Z-score (WZ)<br>follow up: 12 months	N of patients introduction 4-6 n= 372 WZS at 12 months = 0.58 (0.99) - 0.39 (0.95) N of patients introduction at 6 months = 155. WZ at 12 months = 0.25 (0.92) p= 0.01. Association with CF introduction age, fixed for age, sex, maternal age , parity, deprivation score, milk feeding at 3 months			527<br>(1 observational study) [3]	⊕⊕⊕◯<br>MODERATE

	and growth at precedent cut point (Model 3) = 0.01 (-0.06 a 0.07), p= 0.88		
Length Z-score (LZ) follow up: 12 months	Patient CF introduction 4-6 months LZS at 12 months = 0.48 (1,05), 0.23 (1.04). Patient CF introduction at 6 months LZS at 12 months = 0.00 (1,04) p<0,01. Association with CF introduction age fixed for confounding factors (Model 3) 0.04 (-0.01 a 0.11) p= 0,20	(1 observational study) [3]	⊕⊕⊕◯ MODERATE
BMI Z-score (BZ) follow up: 12 months	Introduction CF 4-6 months BMIZ at 12 months 0.42 (0.94) - 0.36 (0.83) CF introduction 6 months, BMIZ at 12 months 0.33 (0.84) p=0.33. Association with CF introduction age fixed for confounding factors (Model 3) -0.02 (-0-08 at 0.05) p= 0.64	(1 observational study) [3]	⊕⊕⊕◯ MODERATE

*** The risk in the intervention group (and its confidence interval (CI) at 95%) is based on the risk assumed in the control group and on the relative outcome of the intervention (and its CI at 95 %).**

CI: Confidence interval; MD: Mean difference

Evaluation of quality of evidence according to GRADE Working Group

High quality: We are very confident that the true effect lies close to that of the estimate of the effect.

Moderate quality: We are moderately confident in the effect estimate: The true effect is likely to be close to the estimate of the effect, but there is a possibility that it is substantially different

Low quality: Our confidence in the effect estimate is limited: The true effect may be substantially different from the estimate of the effect

Very Low quality: We have very little confidence in the effect estimate: The true effect is likely to be substantially different from the estimate of effect.

Explications

a. 2 publications, but from 1 same study
b. for every exposition factor (BF o FF) the study is unique and with low sample numerosity
c. large 95% IC

References

1. 2012, Jonsdottir, et, al.
2. 1996, Bainbridge et al.
3. 2015, Vail. CBGS study.

Table S5b: Risk of overweight/obesity

[Introduction CF at 4-6 months] than [Introduction CF at 6 months] to prevent overweight/obesity at 3-6 years

Patient or population: **prevent overweight/obesity at 3-6 years**

Setting: Outpatient

Intervention: [Introduction CF at 4-6 month

Comparator: [Introduction CF at 6 month]

Outcomes	**Anticipated absolute outcome** * (95% CI)		Relative outcome (95% CI)	№ of participants (studies)	Certainty of evidence (GRADE)	Comments
	**Risk with [Introduction CF at 6 months]**	**Risk with [Introduction CF at 4-6 months]**				
Overweight/obesity at 18 months (S/O 24-36)<br>evaluated with: BMI Z-score<br>follow up: 18 months	109 per 1.000	**141 per 1.000**<br>(40 at 496)	RR 1.30<br>(0.37 at 4.56)	94<br>(1 RCT) [1]	⨁⨁⨁◯<br>MODERATE [a]	
Overweight/obesity at 3 years (S/O 6a)<br>evaluated with: RR (95% IC)<br>follow up: 3 years	2. 463 children, of which 28 (6.1%) were in overweight/obesity condition. The linear regression analysis did not show a statistically significant correlation with the age of introduction of fruit and cereals: coefficient β, respectively = 0,020 (p=0,743) and 0,011 (p=0,828).<br>3. Starting CF at 4 - 6 months (n=427) or at 6 months (n=98). There is no difference in the probability of developing overweight/obesity at 3 years (RR = 0,80; 95%IC = 0,51-1,23)<br>[2,3]			525<br>(2 observational studies) [2,3]	⨁⨁◯◯<br>LOW [b]	

[Introduction CF at 4-6 months] than [Introduction CF at 6 months] to prevent overweight/obesity at 3-6 years

Patient or population: prevent overweight/obesity at 3-6 years

Setting: Outpatient

Intervention: [Introduction CF at 4-6 month

Comparator: [Introduction CF at 6 month]

Outcomes	Anticipated absolute outcome* (95% CI)		Relative outcome (95% CI)	№ of participants (studies)	Certainty of evidence (GRADE)	Comments
	Risk with [Introduction CF at 6 months]	Risk with [Introduction CF at 4-6 months]				

*** The risk in the intervention group (and its confidence interval (CI) at 95%) is based on the risk assumed in the control group and on the relative outcome of the intervention (and its CI at 95 %).**

CI: Confidence interval; RR: Risk ratio

Evaluation of quality of evidence according to GRADE Working Group

High quality: We are very confident that the true effect lies close to that of the estimate of the effect.

Moderate quality: We are moderately confident in the effect estimate: The true effect is likely to be close to the estimate of the effect, but there is a possibility that it is substantially different

Low quality: Our confidence in the effect estimate is limited: The true effect may be substantially different from the estimate of the effect

Very Low quality: We have very little confidence in the effect estimate: The true effect is likely to be substantially different from the estimate of effect.

Explications

a. low sample numerosity

b. the results of the study are coherent with those of other published studies.

References

1. Jonsdottir et al., 2014.
2. Lopes, et. 2016
3. Huh, et al. 2011.

Appendices. References

1. Valerio G, Saggese G, Maffeis C, et al. Diagnosi, trattamento e prevenzione dell'obesità del bambino e dell'adolescente. http://www.siedp.it/files/Doc.ConsensusObesita__2017.pdf
2. Alvisi P, Brusa S, Alboresi S, et al. Recommendations on complementary feeding for healthy, full-term infants. Ital J Pediatr. 2015 Apr 28;41:36.
3. Fewtrell M, Bronsky J, Campoy C, et al. Complementary Feeding: A Position Paper by the European Society for Paediatric Gastroenterology, Hepatology, and Nutrition (ESPGHAN). Committee on Nutrition. J Pediatr Gastroenterol Nutr. 2017 Jan;64(1):119-132
4. Davanzo R, Romagnoli C, and Corsello G. Position Statement on Breastfeeding from the Italian Pediatric Societies. Ital J Pediatr. 2015; 41: 80.
5. NICE guideline 2015. Preventing excess weight gain. nice.org.uk/guidance/ng7
6. Romero-Velardea E, Villalpando-Carrión S, Pérez-Lizaur AB, et al. Consenso para las prácticas de alimentación complementaria en lactantes sanos. Bol Med Hosp Infant Mex. 2016;73(5):338---356
7. American College of Obstetricians and Gynecologists. Breastfeeding Challenges, Obstetrics & Gynecology: February 2021 - Volume 137 - Issue 2 - p e42-e53
8. Qasem W, Fenton T, Friel J. Age of introduction of first complementary feeding for infants: A systematic review. BMC Pediatr. 2015;15:107-18
9. Smith HA, Becker GE. Early additional food and fluids for healthy breastfed full-term infants. Cochrane Database of Systematic Reviews. 2016;8. Art. No.: CD006462
10. USDA, Nutrition Evidence Systematic Review Team and Complementary Feeding Technical Expert Collaborative. Timing of Introduction of Complementary Foods and Beverages and Growth, Size, and Body Composition: A Systematic Review. Pregnancy and Birth to 24 Months Project. Alexandria, VA, U.S. Department of Agriculture, Food and Nutrition Service, Center for Nutrition Policy and Promotion, 2019. Disponibile a https://nesr.usda.gov/project-specific-overview-pb-24-0 (ultimo accesso 31-07-2021)
11. Castenmiller J, de Henauw S, Hirsch-Ernst K-I, et al. EFSA Panel on NDA – Scientific Opinion on the appropriate age range for introduction of complementary feeding into an infant's diet. EFSA J. 2019;17(9):5780
12. Araújo CS, Ribas de Farias Costa P, Alves de Oliveira QueirozV, et al. Age of introduction of complementary feeding and overweight in adolescence and adulthood: A systematic review. Matern Child Nutr. 2019;15:e12796.
13. Brown T, Moore THM, Hooper L. et al. Interventions for preventing obesity in children. Cochrane Database of Systematic Reviews 2019, Issue 7. Art. No.: CD001871.
14. Cordero MJA, Sánchez López AM, Baños NM, et al. Lactancia materna como prevención del sobrepeso y la obesidad en el niño y el adolescente; revisión sistemática. Nutr Hosp. 2015;31(2):606-620
15. Daniels L, Mallan KM, Fildes A, et al. The timing of solid introduction in an 'obesogenic' environment: a narrative review of the evidence and methodological issues Aust NZ J Public Health. 2015; 39:366-73
16. English LK, Obbagy JE, Wong YP, et al. Timing of introduction of complementary foods and beverages and growth, size, and body composition: a systematic review. Am J Clin Nutr. 2019 Mar 1;109(Supplement_7):935S-955S.
17. Grabia M, Markiewicz-Żukowska R, Socha K. Prevalence of Metabolic Syndrome in Children and Adolescents with Type 1 Diabetes Mellitus and Possibilities of Prevention and Treatment: A Systematic Review. Nutrients. 2021 May 23;13(6):1782.
18. Harrison M Wendy Brodribb W. Julie Hepworth J. A qualitative systematic review of maternal-infant feeding practices in transitioning from milk feeds to family foods. Matern Child Nutr. 2017;13:e12360.

19. He S, Stein AD. Early-Life Nutrition Interventions and Associated Long-Term Cardiometabolic Outcomes: A Systematic Review and Meta-Analysis of Randomized Controlled Trials. Adv Nutr. 2021 Mar 31;12(2):461-489.
20. Horta BL, Victora CG. Long-term effects of breastfeeding: a systematic review.III.World Health Organization. 2013 ISBN 978 92 4 150530 7 (NLM classification: WS 125)
21. Horta BL, Loret de Mola C, Victora CG. Long-term consequences of breastfeeding on cholesterol, obesity, systolic blood pressure and type 2 diabetes: a systematic review and meta-analysis. Acta Pædiatrica 2015 104, pp. 30–37
22. Horta BL, Peixoto de Lima N. Breastfeeding and Type 2 Diabetes: Systematic Review and Meta-Analysis. Pediatric Type 2 and Monogenic Diabetes. Current Diabetes Reports (2019) 19:1
23. Kramer MS, Kakuma R. Optimal duration of exclusive breastfeeding. Cochrane Database of Systematic Reviews 2012, Issue 8. Art. No.: CD003517
24. Martin A, Bland RM, Connelly A, et al. Impact of adherence to WHO infant feeding recommendations on later risk of obesity and non-communicable diseases: systematic review. Maternal and Child Nutrition 418 (2016), 12, pp. 418–427
25. Mathew JL. Age of Introduction of Complementary Feeding and Iron Deficiency Anemia in Breastfed Infants. Indian Pediatrics 977 Volume 52__November 15, 2015
26. Mazarello Paes V, Ong KK, Lakshman R. Factors influencing obesogenic dietary intake in young children (0–6 years): systematic review of qualitative evidence. BMJ Open 2015;5:e007396.
27. Moorcroft KE, Marshall JL, and McCormick FM. Association between timing of introducing solid foods and obesity in infancy and childhood: A systematic review. Maternal and Child Nutrition (2011), 7, pp. 3–26
28. Pearce J, Taylor MA, and Langley-Evans SC. Timing of the introduction of complementary feeding and risk of childhood obesity: a systematic review. International Journal of Obesity (2013) 37, 1295–1306
29. Qiao J, Dai LJ, Zhang Q, Ouyang YQ. A Meta-Analysis of the Association Between Breastfeeding and Early Childhood Obesity. J Pediatr Nurs. 2020 Jul-Aug;53:57-66
30. Spill MK, Callahan EH, Shapiro MJ, et al. Caregiver feeding practices and child weight outcomes: a systematic review. Am J Clin Nutr 2019;109(Suppl):990S–1002S.
31. Vail B, Philippa Prentice P, Dunger DB, et al. Age at Weaning and Infant Growth: Primary Analysis and Systematic Review. Pediatr 2015;167:317-24
32. Weng SF, Redsell SA, Swift JA, et al. Systematic review and meta-analyses of risk factors for childhood overweight identifiable during infancy Arch Dis Child 2012;97:1019–1026.
33. Yan J, Liu L, Zhu Y, Huang G, Wang PP. The association between breastfeeding and childhood obesity: a meta-analysis. BMC Public Health. 2014 Dec 13;14:1267.
34. Lopes AF, Rocha EMB, da Silva JPC, et al. Breastfeeding, complementary food introduction and overweight in preschool children. Arch Latinoam Nutr. 2016 Sep;66(3):195-200
35. Huh, S Y, Rifas S, S L, Taveras, E M, et al. Timing of solid food introduction and risk of obesity in preschool-aged children. Pediatrics. 2011;127(3):e544-51
36. Vail B, Philippa Prentice P, Dunger DB, et al. Age at Weaning and Infant Growth: Primary Analysis and Systematic Review. J Pediatr. 2015;167:317-24
37. Bainbridge RR, Mimouni FB, Landi T, et al. Effect of rice cereal feedings on bone mineralization and calcium homeostasis in cow milk formula fed infants. J Am Coll Nutr. 1996;15:383-8
38. Jonsdottir OH, Thorsdottir I, Hibberd PL, Fewtrell MS, Wells JC, Palsson GI, Lucas A, Gunnlaugsson G, Kleinman RE. Timing of the introduction of complementary foods in infancy: a randomized controlled trial. Pediatrics. 2012 Dec;130(6):1038-45.

39. Jonsdottir OH, Kleinman RE, Wells JC, Fewtrell MS, Hibberd PL, Gunnlaugsson G, Thorsdottir I. Exclusive breastfeeding for 4 versus 6 months and growth in early childhood. Acta Paediatr. 2014 Jan;103(1):105-11.
40. Ardic C, Usta O, Omar E, et al. Effects of infant feeding practices and maternal characteristics on early childhood obesity. Arch Argent Pediatr 2019;117(1):26-33 / 26
41. Baldassarre ME, Di Mauro A, Cintoli A. et al. Non-Communicable Chronic Diseases: The Role of Neonatal Characteristics. Iran J Pediatr. 2017 August; 27(4):e9322.
42. Barrera CM, Perrine CG, Li R, et al. Age at introduction to solid foods and child obesity at 6 years. Childh Obes. 2106;12:188-92
43. Bell S, Siau Yi Yew S, Devenish G, et al. Duration of breastfeeding, but not timing of solid food, reduces the risk of overweight and obesity in children aged 24 to 36 months: findings from an Australian cohort
44. Carruth BR, Skinner JD, Houck KS, et al. Addition of Supplementary Foods and Infant Growth (2 to 24 Months). Journal of the American College of Nutrition, Vol. 19, No. 3, 405–412 (2000)
45. Carvalhaes MA, Mialich MA, Rossato SL, et al. Early introduction of food and fluids for healthy infants in 6 months of follow-up. Ann Nutr Metab 2017;71(suppl 2):1–1433
46. Differding MK, Doyon M, Bouchard L, et al. Potential interaction between timing of infant complementary feeding and breastfeeding duration in determination of early childhood gut microbiota composition and BMI. Pediatr Obes. 2020 Aug;15(8):e12642.
47. Gingras V., Aris I.M., Rifas-Shiman S.L., Switkowski K.M., et al. Associations of timing of complementary feeding introduction and adiposity throughout childhood and adolescence. Circulation 2019 139 Supplement 1
48. Grote V, Schiess SA, Closa-Monasterolo R, et al. The introduction of solid food and growth in the first 2 y of life in formula-fed children: analysis of data from a European cohort. Am J Clin Nutr 2011;94(suppl):1785S–Am J Clin Nutr 2011;94(suppl):1785S–93S.
49. Horodynski M.A., Pierce S.J., Reyes-Gastelum D., et al. Feeding Practices and Infant Growth: Quantifying the Effects of Breastfeeding Termination and Complementary Food Introduction on BMI z-Score Growth Velocity through Growth Curve Models. Childhood obesity (Print) 2017 13:6 (490-498)
50. Huus K, Ludvigsson JF, Enskär K, et al. Exclusive breastfeeding of Swedish children and its possible influence on the development of obesity: a prospective cohort study. BMC Pediatrics 2008, 8:42
51. Mannan H. Early Infant Feeding of Formula or Solid Foods and Risk of Childhood Overweight or Obesity in a Socioeconomically Disadvantaged Region of Australia: A Longitudinal Cohort Analysis. Int. J. Environ. Res. Public Health 2018, 15, 1685
52. Martin RM, Kramer RS, Patel R, et al. Effects of promoting longer-term and exclusive breastfeeding on adolescent adiposity, blood pressure, and longitudinal growth trajectories: evidence from the PROBIT cluster-randomized trial. JAMA Pediatr. 2017 July 03; 171(7): e170698
53. Morgen, C. S., Ängquist, L., Baker, J. L., Andersen, A. N., Sørensen, T., & Michaelsen, K. F. (2018). Breastfeeding and complementary feeding in relation to body mass index and overweight at ages 7 and 11 y: a path analysis within the Danish National Birth Cohort. The American journal of clinical nutrition, 107(3), 313–322. https://doi.org/10.1093/ajcn/nqx058
54. Newby RM, and Davies PSW. A prospective study of the introduction of complementary foods in contemporary Australian infants: What, when and why?. Journal of Paediatrics and Child Health 51 (2015) 186–191
55. Olaya G. Buitrago FM, Fewtrell M. Randomised trial testing new complementary feeding guidelines: effects on food consumption and growth at 6 years of age. NUTRITION -Nutrition and health outcomes N-P-185

56. Olaya G, Lawson M, Fewtrell M. Iron Status at Age 6 Months in Colombian Infants Exclusively Breast-fed for 4 to 5 Versus 6 Months. JPGN Volume 64, Number 3, March 2017 (da valutare, inserire nella parte narrativa)
57. Papotsou S, Savva S, Hunsberger M, et al. Timing of solid food introduction and association with later childhood overweight and obesity: The IDEFICS study. Matern Child Nutr. 2018;14:e12471
58. Pluymen LPM, Wijga AH, Gehring U, et al. Early introduction of complementary foods and childhood overweight in breastfed and formula-fed infants in the Netherlands: the PIAMA birth cohort study. European Journal of Nutrition (2018) 57:1985–1993
59. Sandoval Jurado L, Jiménez Báeza MV,Juárez SO, et al. Lactancia materna, alimentación complementaria y el riesgo de obesidad infantil. Aten Primaria. 2016;48(9):572-578
60. Schmidt Morgen C, Ängquist L, Baker JL, et al. Breastfeeding and complementary feeding in relation to body mass index and overweight at ages 7 and 11 y: a path analysis within the Danish National Birth Cohort. Am J Clin Nutr 2018;107:313–322.
61. Seach KA, Dharmage SC, Lowe AJ. Delayed introduction of solid feeding reduces child overweight and obesity at 10 years. International Journal of Obesity (2010) 34, 1475–1479
62. Sirkka O, Vrijkotte T, Halberstadt J, et al. Prospective associations of age at complementary feeding and exclusive breastfeeding duration with body mass index at 5–6 years within different risk groups. Pediatric Obesity 13, 522–529, August 2018.
63. Trovão T, Cavalcante MCV, Rodrigues MC, et al. Determinants of the introduction of early complementary feeding before and after the third month of life: a multinomial analysis. Braz J Med Biol Res. 2020 Nov 18;54(1):e10115.
64. Usheva N, Galcheva S, Cardon G, et al., On Behalf Of The ToyBox-Study Group. Complementary Feeding and Overweight in European Preschoolers: The ToyBox-Study. Nutrients. 2021 Apr 5;13(4):1199.
65. Wells JC, Jonsdottir OH, Hibberd PL, Fewtrell MS, Thorsdottir I, Eaton S, Lucas A, Gunnlaugsson G, Kleinman RE. Randomized controlled trial of 4 compared with 6 mo of exclusive breastfeeding in Iceland: differences in breast-milk intake by stable-isotope probe. Am J Clin Nutr. 2012 Jul;96(1):73-9.
